# Author Correction: Programmable DNA pyrimidine base editing via engineered uracil-DNA glycosylase

**DOI:** 10.1038/s41467-026-79623-1

**Published:** 2026-11-06

**Authors:** Zongyi Yi, Xiaoxue Zhang, Xiaoxu Wei, Jiayi Li, Jiwu Ren, Xue Zhang, Yike Zhang, Huixian Tang, Xiwen Chang, Ying Yu, Wensheng Wei

**Affiliations:** 1 Biomedical Pioneering Innovation Center, Peking-Tsinghua Center for Life Sciences, Peking University Genome Editing Research Center, State Key Laboratory of Protein and Plant Gene Research, School of Life Sciences, Peking University, Beijing, People’s Republic of China; 2 Changping Laboratory, Beijing, People’s Republic of China; 3 Academy for Advanced Interdisciplinary Studies, Peking University, Beijing, People’s Republic of China

**Keywords:** Targeted gene repair, CRISPR-Cas9 genome editing, Genetic engineering

Correction to: *Nature Communications* https://doi.org/10.1038/s41467-024-50012-w, published online 30 July 2024

In the originally published version of this article, errors introduced during preparation of the Source Data file affected that file and the legend to Fig. 1. The experiments shown in Fig. 1e, f and h were performed with two independent biological replicates per condition, and the mean values were correctly used for plotting. However, the corresponding Source Data worksheets incorrectly presented three entries per condition: the two measured values and their calculated mean. The calculated means have now been removed. The legend to Fig. 1, which originally stated *n* = 3 for panels e–h, has been corrected to specify *n* = 2 for panels e, f and h and *n* = 3 for panel g.

The Source Data worksheets for Fig. 2e and g contained data for only 14 of the 16 endogenous sites shown in those panels; the missing data have now been added. The omitted off-target values in row 24 of the Fig. 3b worksheet have also been restored. Two formula-generated columns in the Fig. 2d worksheet did not represent experimental measurements and have been removed. Additional errors in labels, worksheet names and cell formatting have also been corrected.

These errors do not affect the plotted values, results, interpretation or conclusions of the article. No changes to the published figures are required. The legend to Fig. 1 has been corrected in the HTML and PDF versions of the article, and the Source Data file has been replaced with the corrected version.

