# Supplementary material for: Programmable DNA pyrimidine base editing via engineered uracil-DNA glycosylase
Source: Nat Commun. 2024 Jul 30;15:6397. doi: 10.1038/s41467-024-50012-w (PMC11289083; doi:10.1038/s41467-024-50012-w)
Supplement: Supplementary file 1 — Supplementary Information [file 41467_2024_50012_MOESM1_ESM.pdf]

Supplementary Information

Programmable DNA pyrimidine base editing via engineered uracil-DNA glycosylase

Zongyi Yi[1]*, Xiaoxue Zhang[3]*, Xiaoxu Wei[1,2]*, Jiayi Li[1,2]*, Jiwu Ren[2,3], Xue Zhang[3], Yike Zhang[1,2], Huixian Tang[1], Xiwen Chang[2,3], Ying Yu[1], Wensheng Wei[1,3]†

[1]Biomedical Pioneering Innovation Center, Peking-Tsinghua Center for Life Sciences, Peking University Genome Editing Research Center, State Key Laboratory of Protein and Plant Gene Research, School of Life Sciences, Peking University, Beijing, P.R. China.

[2]Academy for Advanced Interdisciplinary Studies, Peking University, Beijing, P.R. China.

[3]Changping Laboratory, Beijing, P.R. China.

*These authors contributed equally to this work.

†Email: wswei@pku.edu.cn (W.W.)

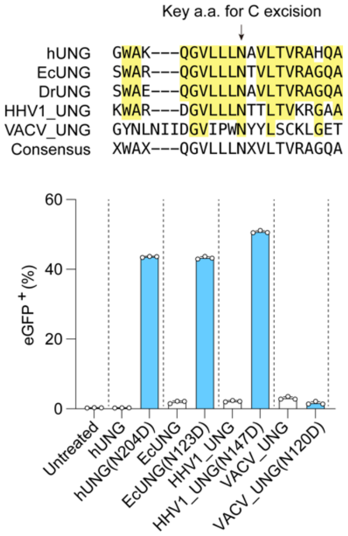

Supplementary Fig. 1 Search more efficient homologous proteins for cytosine excision. Data are presented as mean ± s.d. of n = 3 independent biological replicates. Source data are provided as a Source Data file.

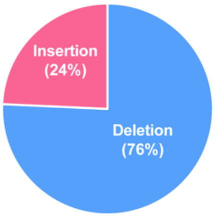

Supplementary Fig. 2 Distribution of indels for DrUNG-based thymine base editors. Source data are provided as a Source Data file.

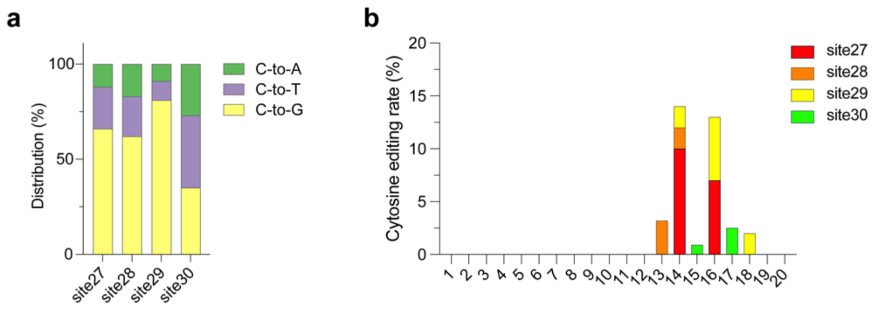

Supplementary Fig. 3 Cytosine base editing is realized based on HHV1_UNG(N147D). **a**, Distribution of editing results of HHV1_UNG(N147D)-based cytosine base editing. **b**, Editing efficiency and position of HHV1_UNG(N147D)-based cytosine base editing. Source data are provided as a Source Data file.

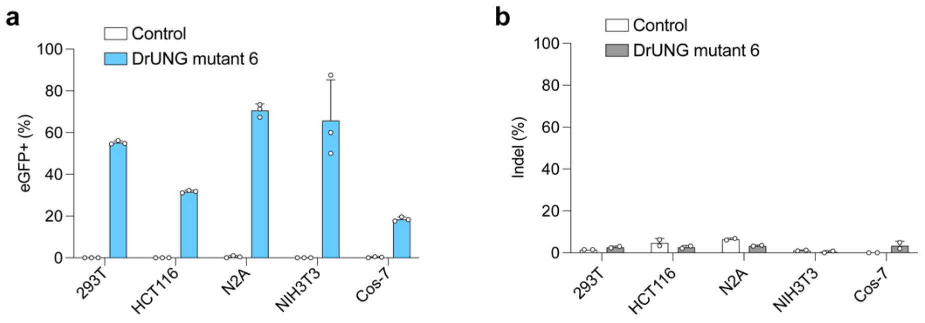

Supplementary Fig. 4 TBEs can achieve thymine base editing in a variety of cell lines. a, Editing efficiency of reporter systems using TBE in multiple cell lines. **b**, Indel of reporter systems using TBE in multiple cell lines corresponding to **a**. Data are presented as mean ± s.d. of n = 3 (**a**) and n=2 (**b**) independent biological replicates. Source data are provided as a Source Data file.

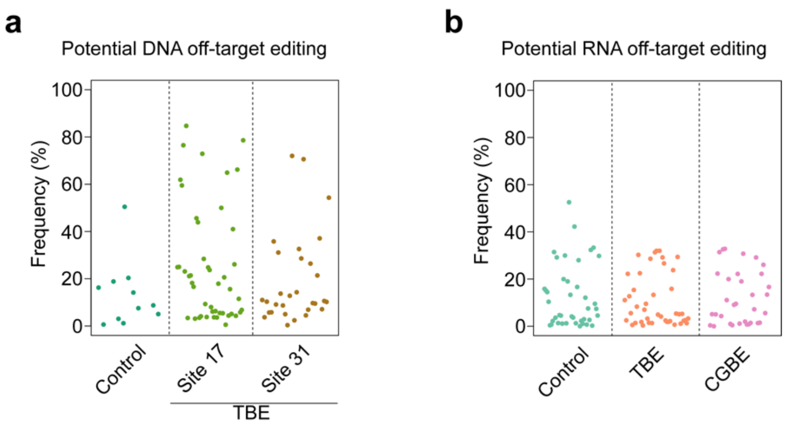

Supplementary Fig. 5 TBEs off-target on the whole genome and transcriptome. a, TBE off-target on the whole genome. b, TBE off-target on the transcriptome. a and b, All data are presented as mean values of n = 3 independent biological replicates. Source data are provided as a Source Data file.

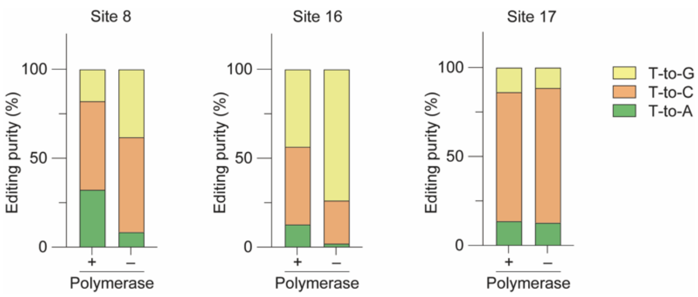

Supplementary Fig. 6 Comparison of editing purity before and after adding polymerase on endogenous site 8, 16 and 17. Data are presented as mean values of n = 3 independent biological replicates. Source data are provided as a Source Data file.

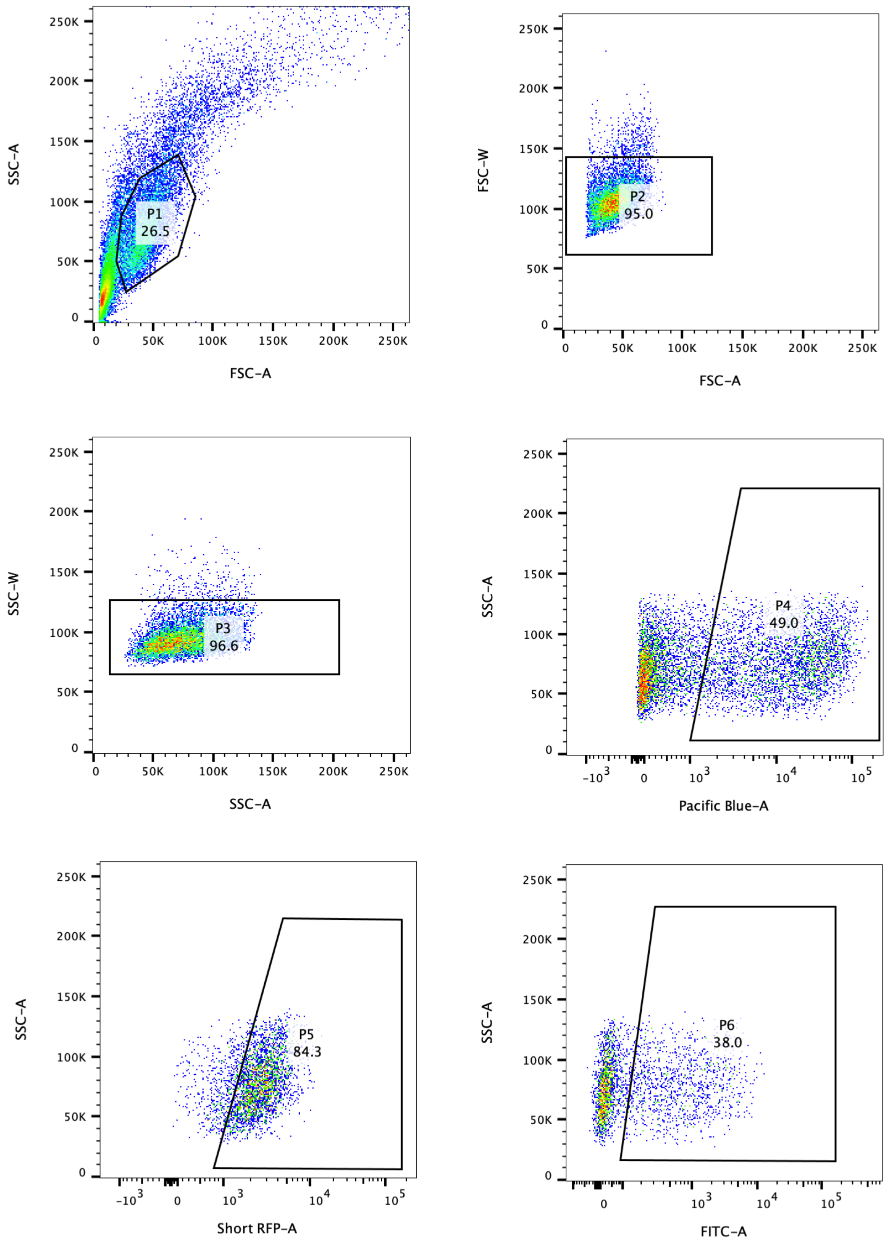

Supplementary Fig. 7 Gating strategies used for FACS on dual fluorescence reporter cell lines. Illustration of FACS (TBE targeted reporter as an example).
