# Supplementary material for: Programmable DNA pyrimidine base editing via engineered uracil-DNA glycosylase
Source: Nat Commun. 2024 Jul 30;15:6397. doi: 10.1038/s41467-024-50012-w (PMC11289083; doi:10.1038/s41467-024-50012-w)
Supplement: Supplementary file 3 — Description of Additional Supplementary Files [file 41467_2024_50012_MOESM3_ESM.pdf]

Description of Additional Supplementary Files

File Name: Supplementary Data 1
Description: The protein and oligo sequences used in this study.

File Name: Supplementary Data 2
Description: The mutation sites of the DrUNG mutants.

File Name: Supplementary Data 3
Description: The sequence of the linkers between DrUNG mutant and nCas9(D10A).

File Name: Supplementary Data 4
Description: The off-target sites of TBEs.

File Name: Supplementary Data 5
Description: The sequence of the sgRNAs and the PCR primers for the target sites.
