# Supplementary material for: Programmable DNA pyrimidine base editing via engineered uracil-DNA glycosylase
Source: Nat Commun. 2024 Jul 30;15:6397. doi: 10.1038/s41467-024-50012-w (PMC11289083; doi:10.1038/s41467-024-50012-w)
Supplement: Supplementary file 5 — Reporting Summary [file 41467_2024_50012_MOESM5_ESM.pdf]

nature portfolio

Corresponding author(s): Wensheng Wei

Last updated by author(s): Jun 14, 2024

Reporting Summary

Nature Portfolio wishes to improve the reproducibility of the work that we publish. This form provides structure for consistency and transparency in reporting. For further information on Nature Portfolio policies, see our Editorial Policies and the Editorial Policy Checklist.

Statistics

For all statistical analyses, confirm that the following items are present in the figure legend, table legend, main text, or Methods section.

n/a	Confirmed	
☐	☒	The exact sample size (*n*) for each experimental group/condition, given as a discrete number and unit of measurement
☐	☒	A statement on whether measurements were taken from distinct samples or whether the same sample was measured repeatedly
☐	☒	The statistical test(s) used AND whether they are one- or two-sided<br>*Only common tests should be described solely by name; describe more complex techniques in the Methods section.*
☒	☐	A description of all covariates tested
☐	☒	A description of any assumptions or corrections, such as tests of normality and adjustment for multiple comparisons
☐	☒	A full description of the statistical parameters including central tendency (e.g. means) or other basic estimates (e.g. regression coefficient) AND variation (e.g. standard deviation) or associated estimates of uncertainty (e.g. confidence intervals)
☐	☒	For null hypothesis testing, the test statistic (e.g. *F*, *t*, *r*) with confidence intervals, effect sizes, degrees of freedom and *P* value noted<br>*Give P values as exact values whenever suitable.*
☒	☐	For Bayesian analysis, information on the choice of priors and Markov chain Monte Carlo settings
☒	☐	For hierarchical and complex designs, identification of the appropriate level for tests and full reporting of outcomes
☐	☒	Estimates of effect sizes (e.g. Cohen's *d*, Pearson's *r*), indicating how they were calculated

Our web collection on statistics for biologists contains articles on many of the points above.

Software and code

Policy information about availability of computer code

Data collection	No software was used.
Data analysis	CRISPResso version 2.2.14 was used for targeted base editing analysis. FastQC, STAR, GATK Haplotypcaller, GATK VariantFiltration, bcftools, ANNOVAR, HISAT2, STRINGTIE and DESeq2 were adopted for genome-wide DNA-sequencing and transcriptome-wide RNA-sequencing analysis.GraphPad Prism 8 was used for basic statistical analysis and graph production.

For manuscripts utilizing custom algorithms or software that are central to the research but not yet described in published literature, software must be made available to editors and reviewers. We strongly encourage code deposition in a community repository (e.g. GitHub). See the Nature Portfolio guidelines for submitting code & software for further information.

Data

Policy information about availability of data

All manuscripts must include a data availability statement. This statement should provide the following information, where applicable:

- Accession codes, unique identifiers, or web links for publicly available datasets
- A description of any restrictions on data availability
- For clinical datasets or third party data, please ensure that the statement adheres to our policy

All data and materials presented in this manuscript are available from the corresponding author (W.W.) upon reasonable request with a completed Material Transfer Agreement. The raw data of off-target analysis generated in this study have been deposited in the China National Center for Bioinformation-National

Genomics Data Center database under accession code subHRA010634. The structure data used in this study are available in the PDB database under accession code 1SSP.

Research involving human participants, their data, or biological material

Policy information about studies with human participants or human data. See also policy information about sex, gender (identity/presentation), and sexual orientation and race, ethnicity and racism.

Reporting on sex and gender	Not applicable.
Reporting on race, ethnicity, or other socially relevant groupings	Not applicable.
Population characteristics	Not applicable.
Recruitment	Not applicable.
Ethics oversight	Not applicable.

Note that full information on the approval of the study protocol must also be provided in the manuscript.

Field-specific reporting

Please select the one below that is the best fit for your research. If you are not sure, read the appropriate sections before making your selection.

☒ Life sciences ☐ Behavioural & social sciences ☐ Ecological, evolutionary & environmental sciences

For a reference copy of the document with all sections, see nature.com/documents/nr-reporting-summary-flat.pdf

Life sciences study design

All studies must disclose on these points even when the disclosure is negative.

Sample size	In this study, targeted base editing of DNA was done with two or three independent biological replicates performed in parallel, and the number of replicates was listed in the text and figure legends. Three independent biological replicates met the needs of the unpaired two-tailed Student's T test, so we performed three independent replicates of the experiment which needed statistics.
Data exclusions	No data were excluded.
Replication	The number of replications is always mentioned in text, methods and figure legends. All attempts at replication were successful.
Randomization	During FACS experiments for sorting transfected cells, all the cells were transfected and sorted via FACS according to the corresponding fluorescence marker, and untreated group and mock group (fluorescence-negative) were conducted for gating.
Blinding	No blinding was performed due to the involvement if several experimentators.

Reporting for specific materials, systems and methods

We require information from authors about some types of materials, experimental systems and methods used in many studies. Here, indicate whether each material, system or method listed is relevant to your study. If you are not sure if a list item applies to your research, read the appropriate section before selecting a response.

Materials & experimental systems

n/a	Involved in the study	
☒	☐	Antibodies
☐	☒	Eukaryotic cell lines
☒	☐	Palaeontology and archaeology
☒	☐	Animals and other organisms
☒	☐	Clinical data
☒	☐	Dual use research of concern
☒	☐	Plants

Methods

n/a	Involved in the study	
☒	☐	ChIP-seq
☐	☒	Flow cytometry
☒	☐	MRI-based neuroimaging

Eukaryotic cell lines

Policy information about cell lines and Sex and Gender in Research

Cell line source(s)	HEK293T (ATCC, CRL-3216) were obtained from C.Zhang's laboratory (Peking University). HCT116 (CCL-247), Neuro-2a (CCL-31), NIH3T3 (CRL-1658), and COS7 (CRL-651) cell lines were maintained in W Wei's laboratory (Peking University). GM06241 (CTCC-001-0896) were purchased from Meisen.
Authentication	STR analysis was used for cell line authentication.
Mycoplasma contamination	All cells tested negative for mycoplasma contamination.
Commonly misidentified lines (See ICLAC register)	No commonly misidentified cell lines were used.

Flow Cytometry

Plots

Confirm that:

☒ The axis labels state the marker and fluorochrome used (e.g. CD4-FITC).

☒ The axis scales are clearly visible. Include numbers along axes only for bottom left plot of group (a 'group' is an analysis of identical markers).

☒ All plots are contour plots with outliers or pseudocolor plots.

☒ A numerical value for number of cells or percentage (with statistics) is provided.

Methodology

Sample preparation	Cells were transfected using PEI transfection reagent according to the supplier's protocols. After 72 hours , cells were digested with trypsin and collected for the following FACS according to the flurorescence marker (mCherry, EGFP or BFP).
Instrument	BD Aria SORP and BD LSRFortessa SORP
Software	BD FACSDiva
Cell population abundance	The fluorescence marker was encoded in the expression plamsids for sorting transfected cells, and about 8x10^5 cells were collected for further process.
Gating strategy	Firstly, the starting cell population were selected according to SSC-A, FSC-A, SSC-W and SSC-H gates. Then fluorescence-positive cell population were determined according to the fluorescence-negative population control (untreated group or mock group).

☒ Tick this box to confirm that a figure exemplifying the gating strategy is provided in the Supplementary Information.
